# Role of Peppermint Oil in Diffuse Esophageal Spasm in the Geriatric Population

**DOI:** 10.7759/cureus.7192

**Published:** 2020-03-06

**Authors:** Swetha Parvataneni, Sireesha Vemuri-Reddy

**Affiliations:** 1 Internal Medicine, Geisinger Health System, Lewistown, USA; 2 Family Medicine, Geisinger Health System, Lewistown, USA

**Keywords:** peppermint oil, diffuse esophageal spasm (des), distal esophageal spasm

## Abstract

Diffuse esophageal spasm, also known as distal esophageal spasm (DES), is a rare motility disorder among the population with symptomatic motility disorders. This disorder is most commonly reported in females, with a median age of 60 years. Multiple therapeutic options have been developed for the treatment of DES. There has been limited research regarding the use of peppermint oil in the treatment of DES. Here, we discuss the interesting case of an elderly female patient who received symptomatic relief from peppermint oil.

## Introduction

Distal esophageal spasm (DES) is a rare, idiopathic motility disorder that predominantly affects females, has a mean age of 60 years, and accounts for approximately 3%-9% of symptomatic patients [1]. Multiple pharmacologic therapies have been developed for the treatment of DES. However, there has been limited research regarding the use of carminatives in DES [2]. The medicinal properties of peppermint oil were first reported by Carl Linnaeus in 1753 [3]. The menthol component in peppermint oil is known to promote relaxation of smooth muscle by dampening the effects of acetylcholine, histamine, 5-hydroxytryptamine, and substance P by inhibiting L-type calcium channels [4]. Other studies have evaluated its use in irritable bowel syndrome, dyspepsia, colonic spasm and headache [5,6] but there is very limited data on the use of peppermint oil in DES. Here, we discuss the interesting case of an elderly female with significant comorbidities who experienced symptom relief from peppermint oil.

## Case presentation

**Figure 1:**
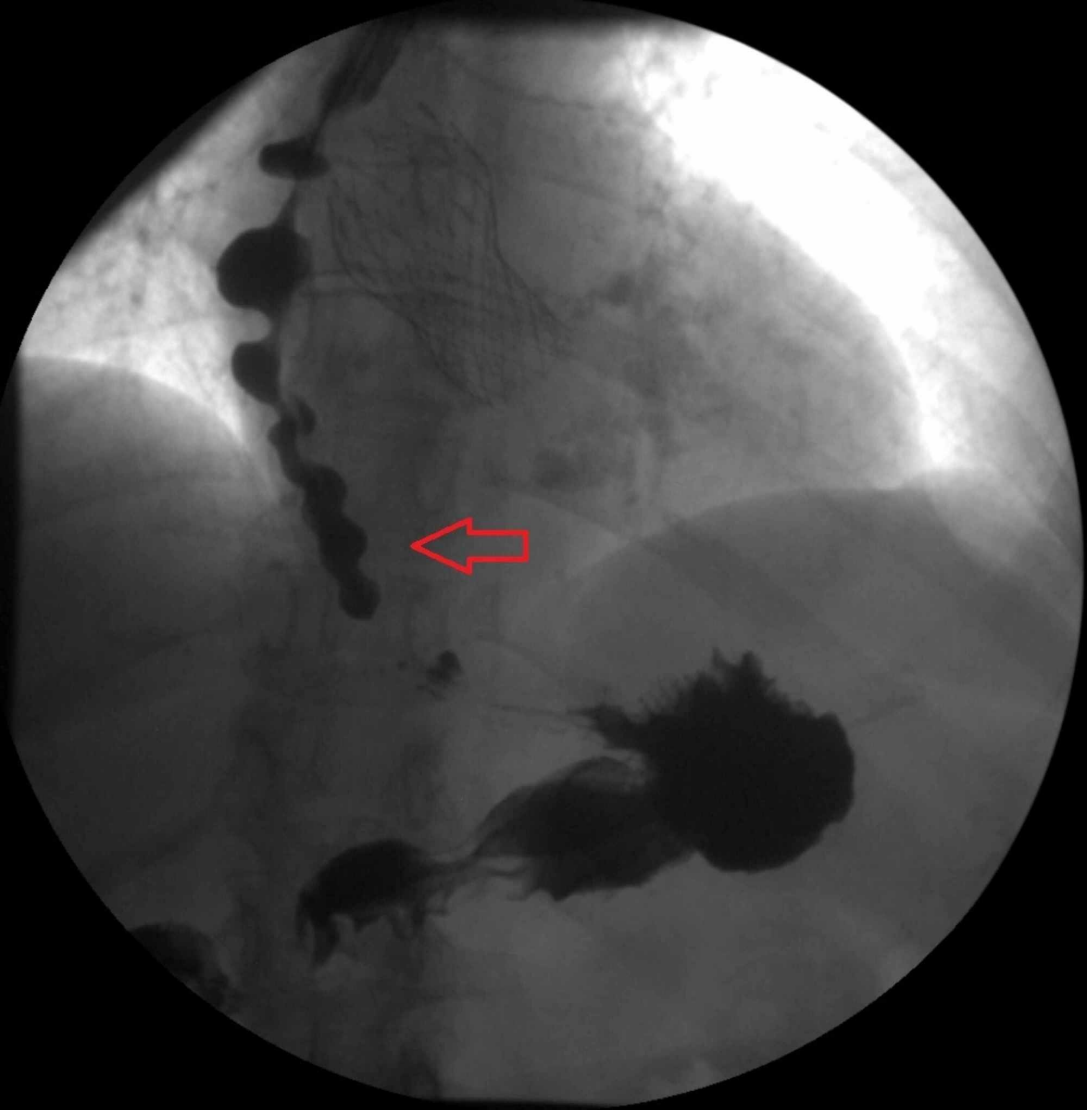
Esophagogram showing diffuse distal esophageal spasm

An 80-year-old female with a past medical history of Alzheimer disease, Parkinson’s disease, brain aneurysm, history of multiple cerebrovascular accidents (CVA) with residual left-sided weakness, history of transcatheter aortic-valve replacement (TAVR), hypertension, hyperlipidemia, and left ventricular diastolic dysfunction was brought to the emergency room (ER) by her daughter due to increased confusion, dysuria, sneezing, swallowing issues, and a reported history of combative behavior against family members. At the appointment with the primary care physician before arrival to the ER, the patient was started on haloperidol to control her agitated behavior. The patient showed improvement with haloperidol as an outpatient. In the ER, the patient was found to have a sodium level of 121 mEq/L, and urinalysis was positive for infection. Given the evidence of urinary tract infection, the patient was managed with intravenous (IV) antibiotics, and her mental status gradually improved. Because of her dysphagia, the patient was given a speech and swallow evaluation and was recommended an esophagogram, which was positive for diffuse DES (Figure 1).

The gastroenterology team was involved in care due to the patient’s dysphagia. Given her multiple comorbidities, a decision was made to treat the DES with peppermint oil rather than nitrates or calcium channel blockers. The patient was started on peppermint oil and discharged to the nursing home. On follow-up, she has reported relief of her dysphagia symptoms with the peppermint oil.

## Discussion

Diffuse esophageal spasm is more appropriately termed DES, as most esophageal spasms involve the lower part of the esophagus. This name was adopted from the Chicago classification of esophageal motility disorders [7,8]. It is a rare, idiopathic motility disorder that predominantly affects females. It is one of the most common causes of non-cardiac-related chest pain after gastroesophageal reflux disease (GERD) [9]. This latter finding was first reported by Osgood in 1889 in a case series, in which all the patients had constricting pain in the epigastrium; Osler named the disorder “oesophagismus” for the pain, and the manometric description was adopted in 1958 [10-12].

DES is thought to be due to a deficiency of nitric oxide, which results in impaired inhibitory innervation and simultaneous contractions of the esophagus. Multiple studies have reported the most common symptoms in patients with DES; these include dysphagia (32%), chest pain (22%), GERD symptoms (20%), cough, hoarseness (13%), and weight loss is occasionally reported [13]. It is very important to rule other cardiac disorders in elderly patients with significant comorbidities before attributing the symptoms to DES. Esophageal manometry is the gold standard for the diagnosis of DES. Earlier guidelines for DES diagnosis included more than 20% of contractions with an amplitude greater than 30 mm Hg with intermittent normal esophageal peristalsis on manometry. Recently, with the introduction of high-resolution manometry, distal latency has been used as a predictor for the diagnosis of DES [14,15].

Treatment for DES is focused on providing symptom relief. The available treatment modalities are classified into endoscopic and pharmacologic options. The endoscopic options include peroral endoscopic myotomy, botulinum toxin, and esophageal dilation while the pharmacologic options include nitrates, tricyclic antidepressants, phosphodiesterase inhibitors, and calcium channel blockers. All of the pharmacologic options have significant side effects. Peppermint oil and its benefits in DES have been discussed in the past, but as yet the research is limited.

An earlier study, published in 2001, reported eight patients with DES who all showed symptom relief and improvement in manometric findings with peppermint oil (p<0.01) [16]. Another study published in 2019 reported the impact of peppermint therapy on dysphagia and non-cardiac chest pain. Thirty-eight patients were included in the study; among these, 63% of patients reported complete symptom resolution, and the remaining patients reported mild-to-moderate symptom improvement. This study involved not only patients with DES but also those with esophagogastric junction outflow obstruction, hypercontractile esophagus, ineffective esophageal motility, and symptomatic patients with normal motility testing [17]. In our case study, we managed an elderly patient with comorbidities who was best treated with peppermint oil treatment in order to avoid side effects that accompany other medications such as nitrates and calcium channel blockers.

## Conclusions

DES is a rare type of motility disorder. Given the side effects associated with available medications for DES, more research is needed on the use of other treatments such as peppermint oil. Given its lack of adverse events, low cost, and easy availability, additional case reports, case series, and prospective trials are needed in the future to better understand the efficacy of peppermint oil.
